# Attitudes Towards Rape and Their Determinants Among Men, Women and Non-Binary People in Poland

**DOI:** 10.1007/s12119-022-10042-2

**Published:** 2022-11-19

**Authors:** Klaudia Olszewska, Przemysław Piotrowski, Bartosz W. Wojciechowski

**Affiliations:** 1 Faculty of Management and Social Communication, Jagiellonian University in Krakow, Krakow, Poland; 2 Institute of Applied Psychology, Jagiellonian University in Krakow, Krakow, Poland

**Keywords:** attitudes, rape, non-binary, authoritarianism, empathy, Poland, ANOVA

## Abstract

The aim of this paper is to present the results of research on attitudes towards rape conducted in a group of 850 adult Poles, including 505 women, 310 men and 35 non-binary people, and to analyze their selected correlates: rape myth acceptance, right-wing authoritarianism and rape empathy. Non-binary people have only recently been included in research as a distinct group and little information can be found in the literature on the characteristics of their attitudes towards social problems. Therefore particular attention was paid to comparing the attitudes towards rape of non-binary people with those of women and men. In analyzing the results, the authors took into account the current socio-political situation in Poland. The results indicate that attitudes toward sexual aggression are related to the type of gender identification. The most positive attitudes towards rape victims among the groups participating in the research are held by non-binary people. Furthermore, attitudes towards rape are determined by rape myths, right-wing authoritarianism and empathy for victims of rape.

## Introduction

Sexual aggression is a worldwide problem present in all societies. According to the National Intimate Partner and Sexual Violence Survey, one in five women in the USA have experienced rape (completed or attempted), and 2.6% of men have been raped (Smith et al., 2018). Data from the National Sexual Violence Resource Center indicate that victims of sexual assault are more likely to be LGBTI than heterosexual (https://www.nsvrc.org/sites/default/files/publications_nsvrc_factsheet_media-packet_statistics-about-sexual-violence_0.pdf). Data on rape victims can only be treated as an estimate, as many victims choose not to report the crime. There are a number of reasons for this: victims are ashamed of what has happened to them, and they fear re-experiencing the trauma, the negative reaction of family and friends, and sometimes revenge from the perpetrator (Hansen et al., 2021; Huemmer et al., 2018; Vidal & Petrak, 2007). Obtaining accurate data on rape victims is also made difficult by the fact that the legal definitions of unacceptable sexual behavior vary from country to country. Social attitudes, beliefs, stereotypes, the level of acceptance of sexual violence and empathy toward victims and perpetrators are also different (Bongiorno et al., 2020; Frese et al., 2004; Henry & Powell, 2014; McKimmie et al., 2014; Stuart et al., 2019).

The statistics of the Polish police show that since the beginning of the 21st century the number of confirmed cases of rape has been constantly decreasing: from 2339 to 2001 to 1034 in 2020 (https://statystyka.policja.pl/st/kodeks-karny/przestepstwa-przeciwko-6/63496,Zgwalcenie-art-197.html). The authors sought to determine whether this positive trend is reflected in the area of social attitudes.

The aim of this paper is to present the results of research on attitudes towards rape conducted in a group of 850 adult Poles, including 505 women, 310 men and 35 non-binary people, and to analyze their selected correlates: rape myth acceptance, right-wing authoritarianism and rape empathy. Due to the fact that non-binary people have only recently been included in research as a distinct group and little information can be found in the literature on the characteristics of their attitudes towards social problems, particular attention was paid to comparing the attitudes towards rape of non-binary people with those of women and men.

### Rape Myths

Martha Burt (1980) was the first to define the term ‘rape myths’ as harmful, stereotypical, or false beliefs about rape, its perpetrators, and their victims. This definition was expanded upon by Lonsway and Fitzgerald (1994), who define rape myths as attitudes and beliefs that are generally false, but widespread and constantly held, that serve to deny and justify male sexual aggression against women. The authors agree that rape can also affect men, but stress that by focusing on male aggression against women, they wish to point out that the overwhelming majority of rape victims are women, while the perpetrators most often turn out to be men, and that there is no analogous set of cultural beliefs that serve to deny and justify the existence of female aggression or male victimization.

Lonsway and Fitzgerald (1994) state three main functions that rape myths serve. First, they serve to deny and trivialize a crime that significantly affects the female population. This is accomplished by shifting the blame for rape from the offender to the victim. Second, one theory that explains victim blaming is the belief in a just world (Lerner & Miller, 1978). This theory posits that people have a need to believe that they get what they deserve and deserve what happens to them - so good people are rewarded and bad people are punished. When some random mishap occurs (in this situation, rape), people with a high belief in a just world tend to view the event as the victim’s fault because it allows them to maintain the worldview that the world is just. This is important because an individual’s recognition that something unjust has happened to someone else assumes that it can happen to them too. As a final function, the authors list oppression and social control of women.

Other functions can also be found in the literature: justification and rejection of male sexual aggression against women (Bohner et al., 2009) and absolving the perpetrator and increasing the responsibility of the victim (Grubb & Turner, 2012).

Some of the most common myths about rape include: rapes happen in public, abandoned places; the victim asks for it herself – by wearing too revealing clothing, drinking alcohol, not showing enough physical resistance - no screaming, no lashing out, no physical injury (McKimmie et al., 2014); the belief that only women are rape victims/no man can be raped, and that rape in a marriage or relationship does not exist (Ayala et al., 2018); the perpetrators of rape are unknown to the victims; they are very often mentally disturbed/very aggressive and have problems with socialization; they come from the margins of society (Stuart et al., 2019); you cannot rape a prostitute (Sprankle, et al., 2018). It is worth noting that the victim is expected to report the rape immediately, be fully cooperative, and be emotional (Masser et al., 2010).

These beliefs are very much ingrained in the social mindset: when a rape victim does not behave in a stereotypical manner, he or she may face negative comments, stigmatization, or even blame. Victims who recognize and internalize rape myths and blame themselves for the rape suffer great psychological distress (Baldwin-White et al., 2016). Some rape victims do not report the matter to the police because they are unsure if the event that occurred was rape or if they are at fault (Baldwin-White et al., 2016; Hayes-Smith & Levett, 2010; King & Roberts, 2011). There is consensus in the literature that acceptance of rape myths is associated with a propensity to engage in sexually aggressive behavior (Chapleau & Oswald, 2010; Edwards et al., 2011; Trottier et al., 2021; Yapp & Quayle, 2018) and negative attitudes toward victims (Hust et al., 2019; McMahon, 2010).

### Right-wing Authoritarianism

Right-wing authoritarianism is a term introduced to psychology by Altemeyer (1981; 1996; 2007), who believes the concept consists of three bundles of attitudes: authoritarian submission, authoritarian aggression, and conventionalism.

The first dimension is expressed in the tendency to conform to authorities that are perceived as recognized and legitimate. It is characterized by widespread acceptance of the actions, statements, and communications of authorities, and a general willingness to follow their instructions without further encouragement. Proponents of right-wing authoritarianism believe that authorities, that is, people who have legal or moral authority over the behavior of others, are to be trusted and respected. Right-wing authoritarians have a low threshold of tolerance for criticism of authority figures because they believe them to be right, while the people who criticize them lack sufficient knowledge, and aim to cause division and destruction. Moreover, in their view, authority figures have the inherent right to decide to what lengths they can go, including the possibility of breaking rules they have made for the rest of society.

Aggression is defined as authoritarian when it is accompanied by the belief that an authority approves of such action or that it will help protect the existing political order. Individuals with high levels of right-wing authoritarianism are proponents of controlling the behavior of others through punishment - thus they are characterized by high levels of punitiveness (Gerber & Jackson, 2015).

Supporters of right-wing authoritarianism are also inclined to blame the victim for the crime, especially when the victim has done something that contradicts their beliefs. Anyone can become a victim of authoritarian aggression, but it most often affects people who behave unconventionally and representatives of minority groups. Supporters of right-wing authoritarianism believe that authorities and authoritarians condone this hostility because these groups threaten the social order.

Conventionalism is expressed in a strong acceptance of, and commitment to, the traditional norms of social life. Thus, most supporters of right-wing authoritarianism believe in divine law and believe that humanity’s conflicts and problems are the result of disobedience to that law. Their attitudes toward sex are strongly tied to religious rules and prohibitions. These attitudes are reflected in conventional perceptions of gender roles (Peterson & Zurbriggen, 2010) and support for the traditional patriarchal family structure and functioning.

### Rape Empathy

Empathy is most often understood as an intrapsychic, complex phenomenon that is based on the ability to understand others’ social roles, attitudes, or opinions; it involves the ability to adjust and harmonize one’s own emotions with those of another person in order to experience that person’s inner experiences, while not identifying with them (Davis, 1996; Vreeke & van den Mark, 2003).

Related to the issue of empathy is a debate about the nature of the concept. The cognitive view of empathy, according to Blair (2005), refers to the ability to understand the feelings and emotions of others and is closely related to theory of mind. Its affective dimension, on the other hand, refers to the experience of emotions, triggered by an emotional stimulus. In the literature, one can find definitions that focus on both the cognitive aspect (Albiero et al., 2009; Hein & Singer, 2008; Stocks et al., 2011), and the emotional aspect (Decety & Michalska, 2010; Van der Weele, 2011; Wispé, 1986). In addition, some definitions include both of these components (Barnett & Mann, 2013; Baron-Cohen & Wheelwright, 2004; Oliveira-Silva & Gonçalves, 2011; Rogers, 1975; Singer & Lamm, 2009).

Rape empathy was defined by Deitz et al. (1982) as an individual’s relative tendency to take the psychological perspective of a rape victim or perpetrator. These authors created the Rape Empathy Scale (RES), an instrument that measures this construct. It assumes that the respondent can feel empathy for either the rape victim or the perpetrator - a high score indicates empathy with the victim, while a low score with the perpetrator.

A slightly different approach was proposed by Smith and Frieze (2003). In their view, rape empathy is a form of generalized empathy occurring in the context of rape that can refer to the victim, the perpetrator, or both. This is because they assume that empathy toward the victim and empathy toward the perpetrator can be independent of each other, as each is measured through different perspectives that may or may not influence each other. This view is also supported by Osman (2011) and Ferrão et al. (2013).

### Aims and Rationale

The purpose of this research is to characterize attitudes toward rape and analyze their personality correlates: rape myth acceptance, right-wing authoritarianism, and rape empathy.

The following hypotheses were formulated:

H1: Attitudes toward rape and their correlates vary between women, men, and non-binary individuals.

H2: Rape myth acceptance, right - wing authoritarianism, empathy relating to rape victims and empathy relating to perpetrators are predictors of the attitudes toward rape.

## Methods

850 individuals, including 505 females, 310 males, and 35 non-binary people, ranging in age from 18 to 62 years (M = 24.1; SD = 6.89), participated in the study. Female participants ranged in age from 18 to 58 years (M = 24.7; SD = 6.65); male participants ranged in age from 18 to 62 years (M = 23.4 SD = 7.4); and non-binary participants ranged in age from 18 to 36 years (M = 22.4; SD = 4.5). 274 of the respondents were students (32.2%), 250 had a high school education (29.4%), 247 had a college education (29.1%), 42 had an elementary education (4.9%), and 37 had a vocational education (4.4%).

Non-binary people are those who do not identify with either the male or female gender. However, this group is internally diverse. Some members do not identify with either gender (*agender, genderless*), others identify with both genders (*pangender, bigender*), experience periodic changes in gender identity (*genderfluid, genderflux*), identify with a gender ‘in-between’ or ‘out of” the binary (*genderqueer, neutrosis*) or feel that they are only partially male or female (*demiboy, demigirl*; see: Matsuno et al., 2021). Although the phenomenon of some people’s identities transcending binary distinctions has a long history (Matsuno & Budge, 2017), it has only begun to be studied scientifically relatively recently. One reason for this is undoubtedly that non-binary individuals challenge the entrenched ontological assumption that there are two genders (Munro, 2007), and that femininity and masculinity are expressed by a set of typical characteristics: those associated with women being motherhood, child-rearing, and beauty, and those linked to men being strength, provision of security, and productivity (Sia Choi et al., 2018). The non-binary group is therefore particularly vulnerable to marginalization and the stress of social exclusion (Richards et al., 2016), which can also entail significant health issues (Gordon et al., 2021). The unique status of this group of people also suggests that their social attitudes towards potentially threatening phenomena (such as rape) will be specific.

This study utilized the Rape Attitude Scale by Olszewska (2021), the Updated Illinois Rape Myth Acceptance Scale (McMahon & Farmer, 2011), the Right-Wing Authoritarianism Scale (Altemeyer, 2007), and two rape empathy tools: the Rape-Perpetrator Empathy Scale (REMP) and the Rape-Victim Empathy Scale (REMV; Smith & Frieze, 2003).

The Rape Attitude Scale, a self-administered tool (Olszewska, 2021), was developed by taking into account the classical three-factor theory of attitudes. It consists of 18 items that are rated on a five-point scale (1 - Strongly disagree, 5 - Strongly agree). The more points the participants score, the more positive their attitudes are towards victims and negative towards perpetrators. To determine the level of reliability of the tool, a pilot study was conducted with 100 Facebook users (77 females, 20 males, and three individuals who did not identify with either gender), aged 19–45 (M = 24.05; SD = 5.09). Cronbach’s alpha was 0.86.

The Polish translation of the Updated Illinois Rape Myth Acceptance Scale by Debowska et al. (2015) was used to measure the level of belief in contemporary rape myths. It contains 19 items to which the respondent refers on a scale from 1 (Strongly Disagree) to 5 (Strongly Agree). The aforementioned authors of the Polish translation examined 319 students and 129 inmates and determined a Cronbach’s alpha level of 0.89.

The Right-Wing Authoritarianism Scale (RWA Scale; Altemeyer 2007) contains 22 statements that the respondent assesses on a scale from − 4 (Strongly Disagree) to 4 (Strongly Agree). This tool was created by taking into account the three factors of right-wing authoritarianism – authoritarian submission, conventionalism, and authoritarian aggression; the Cronbach’s alpha of this tool is 0.90. The RWA Scale version translated into Polish (with the author’s permission) was used in the study. Due to the limitations of conducting the survey online, the respondents referred to the statements on a scale of 1 (Strongly Disagree) to 9 (Strongly Agree). In our own research the Cronbach’s alpha was 0.95.

To measure rape empathy, two instruments were used, created by Smith and Frieze (2003) - the Rape-Perpetrator Empathy Scale (REMP), which measures empathy towards the perpetrator of rape, and the Rape-Victim Empathy Scale (REMV), which relates to empathy for rape victims. Each of these scales consists of 18 items, and the subject responds to the statements given on a scale from 1 (Strongly Disagree) to 5 (Strongly Agree). The Cronbach’s alpha of the tool reported by the authors is 0.84 for REMP and 0.89 for REMV. Our translation into Polish was used in the study (with the authors’ permission). In our study, Cronbach’s alpha was 0.89 for REMP and 0.90 for REMV.

The study was conducted online. The survey sheet was created using Google Form and was arranged in the following order: demographics (age, gender, education), Rape Attitude Scale, Updated Illinois Rape Myth Acceptance Scale, Rape-Perpetrator Empathy Scale, Rape-Victim Empathy Scale, and the Right-Wing Authoritarianism Scale. Participants were recruited through sharing a link to the study on Facebook. Before the study began, participants were informed that the study was anonymous and that they could withdraw at any time.

The analysis of the collected data was divided into two stages. First, we have compared three groups of participants – women, men and non-binary subjects and then we have conducted multivariate regression analysis. Number of participants in the three groups was different (group of non-binary participants was significantly smaller, than women and men), therefore instead of using the parametric Fisher’s ANOVA, we have used the non – parametric equivalent of a one – way ANOVA, that is the Kruskal – Wallis ANOVA. Contrary to the Fisher’s ANOVA, the groups being compared using Kruskal – Wallis ANOVA do not have to be similar in size (Field, 2009; Wilcox, 2005). Accuracy of the *F* statistics is affected by skew if group sizes are not equal, and power of *F* is influenced in case of non – normality (Field, 2009; Wilcox, 2005). Another of the assumptions under which the *F* statistic is reliable, is that the variances in each experimental condition are similar. The Levene’s tests showed, that this condition was not met for the three groups of participants. Therefore comparison of the differences between women, men and non – binary participants was conducted with the Kruskal – Wallis ANOVA, followed by a Dunn - Bonferroni *post – hoc* test.

## Results

The mean score of attitudes towards rape in the sample of 850 participants is 73.52 (*SD* = 9.85), which is well above the halfway mark (54). Moreover, the lowest score achieved by the participants is 28. If, in addition, the standard deviation value is taken into account, the mean score of the subjects’ attitudes towards rape can be considered high (positive towards the victims of the crime). The overall mean score of the subjects’ rape myth acceptance (RMA) is 39.60 (*SD* = 13.81). Mean empathy score towards rape victims in the sample is 72.51 (*SD* = 11.75). The mean score of the REMP scale, measuring the level of empathy towards perpetrators of rape is 43.86 (*SD* = 12.74). In the sample mean score in the right – wing authoritarianism scale was 67.12 (*SD* = 35.54).

Since there were differences between the number of participants in three distinguished groups (*n*_*women*_ = 505, *n*_*men*_ = 310, *n*_*non−binary*_ = 35), in order to verify the first hypothesis, we have performed a one – way, non - parametric Kruskal - Wallis ANOVAs, followed by the Dunn - Bonferroni *post hoc*. Significant differences were found for all variables and in most of the cases between all of the groups. Descriptive statistics and analysis of differences between three sex groups are presented in Table 1.

**Table 1:** Descriptive statistics and analysis of differences between three gender groups

	Women (n = 505)			Men (n = 310)			Non - binary (n = 35)			
	M	SD	Mean rank	M	SD	Mean rank	M	SD	Mean rank	H
SPWZ	76.25	9.09	501.12	68.36	8.83	280.34	79.91	9.74	620.07	178.47*
IRMA	35.88	12.90	353.75	46.59	12.19	563.39	31.31	14.92	239.41	161.12*
REMV	76.08	10.39	502.74	66.11	11.36	287.05	77.68	9.21	537,43	155.95*
REMP	41.05	12.08	371.28	48.66	12.03	518.40	41.91	15.58	384.97	70.01*
RWA	65.61	36.98	407.61	72.57	32.25	480.92	40.71	28.25	192.80	49.92*

*p < .001

The Kruskal - Wallis test revealed that there are statistically significant differences between all three groups in attitudes towards rape (*M*_*women*_ =76.25, *SD*_*women*_= 9.10, *M*_*men*_ =68.36, *SD*_*men*_= 8.83, *M*_*non−binary*_ =79.91, *SD*_*non−binary*_= 9.74; *H*(850) = 178.47, *p* < .001), and *post hoc* Dunn - Bonferroni test showed, that there were differences between all three groups of participants (*χ*^*2*^_*women vs. men*_ = 12.47, p _women vs._ men < 0.0001; χ^2^_women vs. non − binary_=-2.77, p_women vs. men_<0.01; χ^2^_men vs. non – binary_=-7.76, p _men vs. non − binary_<0.001). Results in all of the compared groups differed significantly also for rape myth acceptance (RMA) (*M*_*women*_ =35.88, *SD*_*women*_= 12.90, *M*_*men*_ =46.59, *SD*_*men*_= 12.19, *M*_*non−binary*_ =31.31, *SD*_*non−binary*_= 14.92; *H*(850) = 161.12, *p* < .001), and Dunn - Bonferroni *post hoc* comparison showed, that there were differences between all three groups of participants (*χ*^*2*^_*women vs. men*_ =-11.84, p_women vs._ men < 0.0001; χ^2^_women vs. non − binary_=-2.66, p_women vs. men_<0.01; χ^2^_men vs. non – binary_=-7.40, p_men vs. non − binary_<0.001); as well as for the right – wing authoritarianism scale (*M*_*women*_ =65.61, *SD*_*women*_= 36.98, *M*_*men*_ =72.57, *SD*_*men*_= 32.25, *M*_*non−binary*_ =40.71, *SD*_*non−binary*_= 28.25; *H*(850) = 49.92, *p* < .001), in this case *post hoc* comparison (Dunn - Bonferroni) also confirmed statistically significant differences within all three pairs of participants (*χ*^*2*^_*women vs. men*_ =-4.14, p_women vs._ men < 0.0001; χ^2^_women vs. non − binary_=-5.01, p_women vs. men_<0.001; χ^2^_men vs. non – binary_=-6.58, p_men vs. non − binary_<0.001).

For the empathy towards rape victims (REMV) scale there were differences between the groups (*M*_*women*_ =76.08, *SD*_*women*_= 10.39, *M*_*men*_ =66.11, *SD*_*men*_= 11.36, *M*_*non−binary*_ =77.68, *SD*_*non−binary*_= 9.21; *H*(850) = 155.95, *p* < .001), but in this case *post hoc* comparison conducted with the Dunn - Bonferroni test confirmed only differences between two pairs of participants, that is women vs. men, and non – binary participants vs. men (*χ*^*2*^_*women vs. men*_ =-12.18, p_women vs._ men < 0.001; χ^2^_women vs. non − binary_=-0.81, p_women vs. men_=0.42; χ^2^_men vs. non – binary_=-5.72, p_men vs. non − binary_<0.001). Study showed significant differences between the level of the empathy for the perpetrator (REMP) among the compared groups (*M*_*women*_ =41.05, *SD*_*women*_= 12.08, *M*_*men*_ =48.66, *SD*_*men*_= 12.03, *M*_*non−binary*_ =41.91, *SD*_*non−binary*_= 15.58; *H*(850) = 70.01, *p* < .001) but similar to empathy towards rape victims (REMV) results, there were no statistically significant differences between women and non – binary participants in level of the empathy for the perpetrator (REMP) (*χ*^*2*^_*women vs. men*_ =-8.31, p_women vs._ men < 0.001; χ^2^_women vs. non − binary_=-0.32, p_women vs. men_=0.75; χ^2^_men vs. non – binary_=-3.05, p_men vs. non − binary_<0.01).

In order to verify the second hypothesis, we have conducted multiple linear regression. A significant regression equation was found (*F* (4,845) = 579.50, *p* < .001) with an *R*^*2*^ of 0.733. Participants’ predicted attitudes toward rape are equal to 81.23 − 0.36 (IRMA, rape myth acceptance; *t* = -18.88, *p* < .001) + 0.21 (REMV, empathy relating to rape victims; *t* = 12.35, *p* < .001) − 0.10 (REMP, empathy relating to perpetrators; *t* = -6.46, *p*<) − 0.05 (RWA, right – wing authoritarianism; *t*=-8.53, *p* < .001).

## Discussion

The purpose of this study was to characterize attitudes toward rape and examine their correlates – rape myth acceptance, right-wing authoritarianism, and rape empathy.

The hypothesis that attitudes toward rape and their correlates differ across female, male, and non-binary individuals was confirmed. In our study, women’s attitudes toward rape were more positive than men’s attitudes. These findings are consistent with studies available in the literature. Rape predominantly affects women; they are much more likely than men to be victimized by this crime, which may influence their more positive perceptions of victims, greater knowledge of the subject, and less stereotypical perceptions of rape. In a study by Hockett et al. (2009), women scored statistically lower on the Attitudes Toward Rape Victims Scale, which in this study implied more positive attitudes toward rape victims; additionally, they also scored lower on the Rape Myth Acceptance Scale. Similar results were obtained by McKimmie et al. (2014): in their study, women exhibited more positive attitudes toward the victims, were more confident that a crime had occurred, and had less positive attitudes toward the perpetrators than men. In the study conducted by Klement et al. (2019), women also displayed more positive attitudes toward victims and more negative attitudes toward offenders than men.

One can only speculate to what extent these attitudes are derived from the widespread feeling among Polish women that they are a group whose rights and freedoms have been gradually restricted since the right-wing Law and Justice government took power in 2015. Since 2016, when in September the Polish Parliament rejected a civic bill liberalizing abortion law, Ogólnopolski Strajk Kobiet (All-Poland Women’s Strike; OSK) has been active in Poland. It opposes the tightening of anti-abortion laws and fights for women’s rights in other spheres, such as the work environment. OSK organized mass social protests and demonstrations between 2016 and 2021.

Furthermore, the results indicate that the most positive attitudes towards rape victims and negative towards rape perpetrators among the groups participating in the research are held by non-binary people. Positive attitudes of non-binary people towards the victims of rape may be an expression of identification with broadly understood marginalized groups and groups at risk of victimization. Non-binary people often face attacks in their local communities and sphere of online contacts. They also receive little support from those closest to them, feel misunderstood and isolated (Aparicio-Garcia, et al., 2018; Frohard-Dourlent et al., 2017; Scandurra et al., 2019). Prejudice against gender nonconformists is particularly strong in Poland (Konopka et al., 2020; Świder & Winiewski, 2017). The public debate about the rights of sexual minorities is very intense, and representatives of the LGBTI community often feel attacked and discriminated against, especially by those with right-wing views. Moreover, activism for the equal treatment of all types of gender identity is often portrayed as part of a ‘gender conspiracy’, that is, alleged behind-the-scenes activities that seek to destroy traditional values, Polish national culture, and the foundations of the Catholic faith (Marchlewska et al., 2019). One of the manifestations and effects of this type of narrative was the creation of so-called LGBTI-free zones, which meant some Polish municipalities passed homophobic laws that discriminated against non-binary people and their families (Noack, 2019). These manifestations of homophobic discrimination were condemned in the European Parliament Resolution of 18 December 2019 on discrimination against and incitement to hatred of LGBTI people in the public sphere, including LGBTI-free zones (2019/2933(RSP).

The second hypothesis has also been confirmed: attitudes towards rape are determined by rape myths, right-wing authoritarianism and empathy for victims of rape.

Non-binary individuals have the lowest level of acceptance of rape myths. This result can probably be attributed to the sensitization of non-binary people to the stereotyping and stigmatization that they still face in the Polish reality, and which was highlighted in the Memorandum on the stigmatization of LGBTI people in Poland by the Commissioner for Human Rights (Council of Europe, 3 December 2020; see: https://rm.coe.int/memorandum-na-temat-stygmatyzacji-osob-lgbti-w-polsce/1680a08bb4). LGBTI people have been frequently attacked and subjected to acts of symbolic violence during the last few years of the right-wing Law and Justice party’s rule in Poland. As Jakubowska-Branicka (2020, pp. 164-5) puts it:

“The reality created by the Law and Justice party abounds in numerous dichotomous divisions: citizens holding political views and beliefs different from those supported by the party are labelled the “worse sort”; new “enemies” are constantly pointed out – refugees, gender, and non-heteronormative people; representatives of the opposition are often defined as “traitors”, which is unthinkable in a democratic discourse”.^1^

Non-binary people scored lowest on the RWA Scale. Right-wing authoritarianism is expressed by, among other things, a commitment to traditional values and a conservative view of gender roles. Thus, there is an assumption that individuals who exhibit high levels of right-wing authoritarianism tend to stereotype perceptions of rape and assign blame to the victim (Canto et al., 2020, 2021). By simply belonging to non-binary group, its members stand in opposition to the values of right-wing authoritarianism - respect for traditional social values, and entrenched thinking about gender binarity and assigned social roles for men and women.

There were no statistically significant differences between women and non - binary participants in the level of empathy for the rape victims (REMV) and rape perpetrators (REMP). Emotional relation to the object is a very important element of attitudes held. Thus, it is not surprising that people who have positive emotions toward rape victims will also be more likely to reflect on their difficult psychological situation and relate it to themselves, thus empathizing - both cognitively and affectively. Empathy toward the perpetrator, on the other hand, is associated with an understanding and experience of rape situation that devalues the victim. Apparently, in the context of rape, the psychological situation of women and non-binary people is similar. People belonging to both groups are probably characterized by a heightened level of anxiety in connection with a possible attack - both in the sphere of real and online contacts.

## Limitations and Future Research

One of the main limitations of the studies presented above is the form in which they were conducted – due to the epidemiological situation in the country as a result of the COVID-19 pandemic, it was decided to conduct the study using the Internet. The tools used in this research do not have Polish standardization - both the Right-Wing Authoritarianism Scale and REMV and REMP were translated from English for the purposes of this research, which could potentially have affected the results. The participants in the study ranged in age from 18 to 62, but the average was 24.1, meaning that the sample consisted mainly of young adults. Moreover, the study only included people with access to the Internet, including Facebook, who were computer proficient enough to handle filling out the Google Form. This means that the study involved a group of people that may not be representative of the entire Polish population.

Only 35 non-binary participants took part in the present study; the size of this group was much smaller than the size of the other groups in this study. Moreover, Levene’s test revealed differences in variance. Therefore our statistical analysis aiming at group comparisons had to be limited to the non - parametrical ANOVA (Kruskal – Wallis test) and the ability to draw clear conclusions was limited. Non-parametric tests have lower statistical power than parametric tests. This may explain why we did not find statistically significant differences between women and non-binary participants in the level of empathy towards rape victims (REMV) and the level of the empathy for the perpetrator (REMP).

On the other hand, the number of non-binary people in the total study population is 4.1%, which is probably similar to the percentage in the Polish population. This percentage is difficult to determine unequivocally. In July 2021, the Polish Ombudsman pointed out that non-binary people do not have the opportunity to demonstrate their gender identity in the National Census (https://bip.brpo.gov.pl/pl/content/rpo-gus-spis-powszechny-osoby-transplciowe-niebinarne). There is also not much scientific research in Poland on the psychological situation of this group of people (Grabski et al., 2021). In a Dutch study, ambivalent identification with gender was found in 4.6% of men and 3.2% of women (Kuyper & Wijsen, 2014). In Brazil, the number of non-binary individuals was estimated at 1.19% (Spizzirri et al., 2021).

The results of the analyses conducted encourage further, more detailed and extensive research on attitudes toward rape. It would be optimal for the research to take place under more controlled conditions, and for the groups participating in the study to be as equal as possible, with a higher mean age.

Further research with the inclusion of a non-binary group would seem to be of particular interest (Scandurra et al., 2019). The psychosocial situation of non-binary people should be monitored all the more carefully, as the existence of anti-LGBTI attitudes in Poland, marginalization and stereotypical representation of this social group can still be observed (Chojnicka, 2015; Mole et al., 2021). Actions for the inclusion of non-binary people are consistent with one of the goals of the UN document *Transforming our World: the 2030 Agenda for Sustainable Development* (2015), which is to “Promote peaceful and inclusive societies for sustainable development, provide access to justice for all and build effective, accountable and inclusive institutions at all levels”.

## Footnotes

1 Translated by the authors.
